# Dual Innervation of Neonatal Merkel Cells in Mouse Touch Domes

**DOI:** 10.1371/journal.pone.0092027

**Published:** 2014-03-17

**Authors:** Jingwen Niu, Anna Vysochan, Wenqin Luo

**Affiliations:** Department of Neuroscience, Perelman School of Medicine, University of Pennsylvania, Philadelphia, Pennsylvania, United States of America; University of Vienna, Max F. Perutz Laboratories, Austria

## Abstract

Merkel cell-neurite complexes are specialized mechanosensory end organs that mediate discriminative touch sensation. It is well established that type I slowly adapting (SAI) mechanoreceptors, which express neural filament heavy chain (NFH), innervate Merkel cells. It was previously shown that neurotrophic factor NT3 and its receptor TrkC play crucial roles in controlling touch dome Merkel cell innervation of NFH^+^ fibers. In addition, nerve fibers expressing another neurotrophic tyrosine receptor kinase (NTRK), Ret, innervate touch dome Merkel cells as well. However, the relationship between afferents responsive to NT3/TrkC signaling and those expressing Ret is unclear. It is also controversial if these Ret^+^ fibers belong to the early or late Ret^+^ DRG neurons, which are defined based on the co-expression and developmental dependence of *TrkA*. To address these questions, we genetically traced Ret^+^ and TrkC^+^ fibers and analyzed their developmental dependence on *TrkA*. We found that Merkel cells in neonatal mouse touch domes receive innervation of two types of fibers: one group is Ret^+^, while the other subset expresses *TrkC* and *NFH*. In addition, Ret^+^ fibers depend on *TrkA* for their survival and normal innervation whereas NFH^+^ Merkel cell innervating fibers are almost unaltered in *TrkA* mutant mice, supporting that Ret^+^ and NFH^+^/TrkC^+^ afferents are two distinct groups. Ret signaling, on the other hand, plays a minor role for the innervation of neonatal touch domes. In contrast, Merkel cells in the glabrous skin are mainly contacted by NFH^+^/TrkC^+^ afferents. Taken together, our results suggest that neonatal Merkel cells around hair follicles receive dual innervation while Merkel cells in the glabrous skin are mainly innervated by only SAI mechanoreceptors. In addition, our results suggest that neonatal Ret^+^ Merkel cell innervating fibers most likely belong to the late but not early Ret^+^ DRG neurons.

## Introduction

Touch sensation, which is mediated by primary mechanosensory neurons, is critical for our daily life and social interactions. The mammalian mechanosensory neurons are classified into different types based on their anatomical features and physiological properties. One main type of mechanosensory neurons is the Aβ low-threshold mechanoreceptors (Aβ LTMR) [1], [2], [3], which are large-diameter, NFH^+^, have highly myelinated axons, and innervate morphologically specialized mechanosensory end organs. Aβ LTMR can be further divided into either rapidly adapting (RA) or slowly adapting (SA) mechanoreceptors based on their adaptation properties to sustained mechanical stimuli [4]. RA mechanoreceptors usually fire action potentials at the onset and offset of a sustained mechanical stimulus while SA mechanoreceptors fire action potentials continuously.

The Merkel cell-neurite complex is one of the best known types of mechanosensory end organs [2], [5], [6]. Each complex is composed of specialized epidermal cells, Merkel cells, and innervating somatosensory fibers. Merkel cells are clustered in skin regions that are specialized for high tactile acuity, such as fingertips, whisker follicles, and touch domes of hairy skin [7]. Since afferents displaying type I SA mechanoreceptor properties (SAI) usually innervate skin regions that are enriched with Merkel cells [5], [8], it is well accepted that the Merkel cell-neurite complexes are the “SAI” mechanoreceptors.

Neurotrophic factor signaling plays critical roles in development of Merkel cell-neurite complexes [9]. In mouse whisker pad, Merkel cell innervating trigeminal ganglion (TG) neurons express neurotrophic tyrosine kinase receptors (NTRK) TrkA and TrkC [10], [11], [12]. The ablation of *TrkA* leads to a substantial reduction in the number of innervating neurites and Merkel cells, with some Merkel cell-neurite complexes surviving to adulthood. Loss of *NGF*, the TrkA ligand, produces a similar, but less severe phenotype [10]. On the other hand, loss of *TrkC* or *NT3* leads to a more severe deficit, with even fewer nerve endings and Merkel cells present at birth, and nearly all nerve endings and Merkel cells disappear by postnatal day 7 (P7). Moreover, the double knockout of *TrkA* and *TrkC* results in a complete loss of Merkel cells in the whisker pad [11]. These results suggest that two types of Merkel cells innervating nerve fibers may exist in the mouse whisker pad: one type which requires NGF/TrkA signaling, and the other type which depends on NT3/TrkC signaling for their survival and innervation. In touch domes of back hairy skin, the development of Merkel cell-neurite complexes highly depends on NT3/TrkC signaling [13], [14], [15], [16]. Nevertheless, it remains to be determined whether Merkel cells in touch domes or glabrous skin also receive other types of nerve fiber innervation.

Ret is another NTRK that plays critical roles in controlling development of somatosensory neurons. *Ret* is expressed in approximately 60% of adult mouse dorsal root ganglion (DRG) neurons, which are broadly divided into two main groups based on their development process [17], [18]. Most Ret^+^ DRG neurons are small to medium-diameter nociceptors and come from TrkA^+^ precursors. They depend on TrkA signaling for many prenatal developmental processes, including survival, gene expression, and axonal growth. *Ret* is not highly expressed in these neurons until embryonic day 13.5 (E13.5) or beyond [17], [18]. A distinct, small population of Ret^+^ DRG neurons is born early, expresses *Ret* prior to E13.5, does not express *TrkA*, and has large diameter soma. These large diameter Ret^+^/TrkA^–^ neurons are defined as the early Ret^+^ neurons [17], [18], [19], [20]. Recently, one study suggested that the early Ret^+^ neurons develop into both RA and SAI mechanoreceptors that innervate the hairy skin [19]. The authors found that Ret^+^ nerve fibers innervate touch domes and form lanceolate endings around the hair follicle (one type of RA mechanosensory end organs) in neonatal mice. In addition, they showed that NFH^+^ fibers innervating Merkel cells and hair follicles are decreased in the *Ret* null mice. On the other hand, our previous study using genetic tracing of the early Ret^+^ DRG neurons found that they innervate all types of RA mechanosensory end organs but not Merkel cells, suggesting that the early Ret^+^ DRG neurons develop into RA mechanoreceptors specifically [20]. This discrepancy raises the question of whether or not the early Ret^+^ DRG neurons develop into SAI mechanoreceptors.

In this paper, we used a combination of neurotrophic factor dependence analysis and genetic tracing of Ret^+^ and TrkC^+^ fibers to address two questions: 1) if Merkel cells in the touch domes and glabrous skin receive more than one type of nerve fiber innervation; and 2) if Merkel cell innervating Ret^+^ fibers are the early Ret^+^ population. We found that Merkel cells in the mouse back hairy skin are innervated by two different types of fibers. One group is Ret^+^ and depends on *TrkA* for their neonatal survival/Merkel cell innervation. The other group of fibers is NFH^+^/TrkC^+^, which are independent of *TrkA* for their survival and Merkel cell innervation. In the glabrous skin, Merkel cells are mostly contacted by NFH^+^/TrkC^+^ but Ret^−^ afferents. Taken together, our results reveal that Merkel cells in the neonatal trunk hairy skin but not glabrous skin receive dual innervation. In addition, our results suggest that Ret^+^ fibers, which innervate neonatal Merkel cell, most likely belong to the late but not early Ret^+^ DRG neurons.

## Materials and Methods

### Ethics Statement

Mice were raised in a barrier facility in the Hill Pavilion, University of Pennsylvania. All procedures were conducted according to animal protocols approved by Institutional Animal Care and Use Committee (IACUC) of the University of Pennsylvania and the National Institutes of Health guidelines.

### Mouse strains

Mice used in this paper were described previously: *Ret^CFP/+^* [20]_ENREF_1, *TrkA^+/^* ^−^ [21], and *TrkC^GFP^*_ENREF_3 [22], [23].

### Tissue preparation and Immunohistochemistry

Mice were euthanized with CO_2_, transcardially perfused with PBS/4% paraformaldehyde (PFA). Back hairy skin tissue (de haired with Nair) and footpad of hind paw were dissected and post-fixed with 4% PFA for 2 hours at 4°C, and cryo-protected in 30% sucrose in 1XPBS overnight. Frozen sections of 40μm were cut using a Leica CM1950 cryostat. Immunostaining of sections, and whole mount hairy skin were performed as described previously [24], [25]. Antibodies used are as follows: rat anti-cytokeratin 8 (DSHB, TROMA1, 1∶20), goat anti-TrkC (R&D, AF1401, 1∶1000), chicken anti-GFP (Aves, GFP-1020, 1∶1000), rabbit anti-GFP (Invitrogen, A-11122, 1∶2000), chicken anti-NFH (Aves, NF-H, 1∶1000), rabbit anti-NFH (Sigma, N4142, 1∶1000), and Alexa Fluorescent conjugated Goat or Donkey secondary antibodies (Invitrogen or Jackson IR, 1∶500-1000).

### Image acquisition and Quantification

Fluorescent images were acquired on a Leica SP5II confocal microscope. We conducted serial optical sections (∼1μm Z stack) for tissue samples. For P0 stainings, we consider a nerve fiber innervating Merkel cell when it overlaps with a Merkel cell at the same optical section. In adult, the nerve fibers innervating Merkel cells have more mature morphologies, which usually expand at the terminal and form “synapse like” structures contacting Merkel cells. We consider a nerve fiber which displays such morphology to innervate Merkel cells. We counted the total number of Merkel cells within a touch dome (or in glabrous skin sections) and the number of Merkel cells receiving a given type of fiber innervation. We then normalized the data as the percentage of Merkel cell receiving innervation. This is because the number of Merkel cells per touch dome varies depending on the section angle while Merkel cells in the glabrous skin have differential distribution in different skin regions [7]. We usually count 6 to 10 touch domes or 6 to 10 glabrous skin sections per mice and use at least three mice per genotype for quantification and statistical analysis. Cell number counting was performed using ImageJ, column graphs and scatter plots were generated in GraphPad Prism 5. All error bars are ± standard error of the mean (s.e.m.). Mixed model analysis in SAS was used to compare the significance between different genetic backgrounds.

## Results

### Mouse neonatal touch domes are innervated by Ret^+^ and NFH^+^ fibers

**Figure 1:**
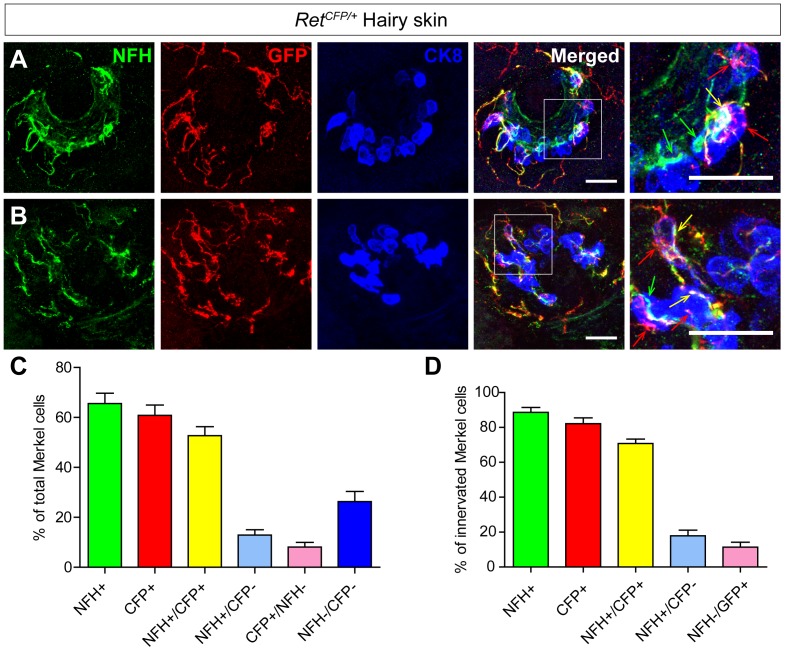
Neonatal touch dome Merkel cells are innervated by Ret^+^ and NFH^+^ fibers. A-B, Triple staining of the back hairy skin of P0 *Ret^CFP +/^* ^−^ mice with antibodies against CK8, GFP and NFH. Two representative examples of touch domes are shown in A and B. CFP^+^ Merkel cell innervating fibers are indicated by red arrows, NFH^+^ fibers are indicated by green arrows, and the overlap spots of Ret^+^ and NFH^+^ fibers are indicated by yellow arrows. C, Quantification of the percentages of Merkel cells innervated by Ret^+^ and NFH^+^ fibers out of total Merkel cells in the touch domes. D, Quantification of the percentages of different fiber innervation out of the innervated Merkel cells in the touch domes. Scale bar, 20 μm. n = 3 animals. See also Table 1.

To label Ret^+^ fibers that innervate Merkel cells, we used a knockin mouse line, *Ret^CFP/+^* [20], in which a cyan fluorescent protein (CFP) is expressed from the *Ret* allele and thus labels all Ret^+^ cells. We examined Merkel cell innervation in the P0 back hairy skin using antibodies against cytokeratin 8 (CK8), green fluorescent protein (GFP), and NFH. CK8 stains Merkel cells, GFP antibody recognizes CFP, and NFH labels the highly myelinated fibers which are presumably SAI mechanoreceptors (Figure 1A-B). We found that both CFP^+^ and NFH^+^ fibers innervate Merkel cells in the P0 mouse back hairy skin. 73.71±4.09% of total Merkel cells receive at least one type of innervation: 60.81±4.19% of total Merkel cells are innervated by CFP^+^ fibers (red arrows), while 65.59±4.11% of total Merkel cells are innervated by NFH^+^ (green arrow) fibers (Figure 1C, and Table 1). Within the innervated Merkel cells, most of them (70.78±2.48% of the innervated Merkel cells, which equals to 52.68±3.60% of the total Merkel cells, (Figure 1C-D)) receive both CFP^+^ and NFH^+^ fibers (yellow arrows) (Figure 1A-B), while a small percentage of Merkel cells receive only CFP^+^ or NFH^+^ innervation (Figure 1C).

**Table 1:** Touch dome Merkel cell innervation in P0 *Ret^CFP/+^* and mutant hairy skin.

Genotype	# of TD	NFH^+^ TD (%)	GFP^+^ TD (%)	MC per TD	NFH^+^ MC (%)	GFP^+^ MC (%)	NFH^+^/GFP^+^ MC (%)	# of animal
*Ret^CFP/+^*	19	19 (100%)	19 (100%)	20.32±1.381	13.37±1.188 (65.59±4.110%)	12.58±1.325 (60.81±4.185%)	10.79±1.016 (52.68±3.595%)	N = 3
*Ret^CFP/CFP^*	27	27 (100%)	27 (100%)	17.85±2.008	10.85±1.193 (62.95±2.931%)	8.741±1.055 (51.00±3.378%)	7.407±0.8791 (44.23±3.563%)	N = 3
				n.s. P = 0.3601	n.s. P = 0.5723	n.s. P = 0.1635	n.s. P = 0.1121	

TD, touch dome; MC, Merkel cell; n.s., no significance

Interestingly, even for Merkel cells innervated by both CFP^+^ and NFH^+^ fibers, we noticed that some CFP and NFH signals do not overlap with each other (Figure 1A-B). These results suggest two possibilities: 1) Ret^+^ and NFH^+^ fibers belong to the same population. Some of their staining signals don’t completely overlap because CFP, which genetically labels the Ret^+^ neurons, and NFH have differential subcellular location; and 2) Ret^+^ and NFH^+^ fibers are two different types of afferents. However, their staining signals overlap to a large extent under the light microscope because they travel closely in the same nerve bundle and only defasciculate when they get close to the Merkel cells.

### Touch dome innervating NFH^+^ fibers are TrkC^+^

**Figure 2:**
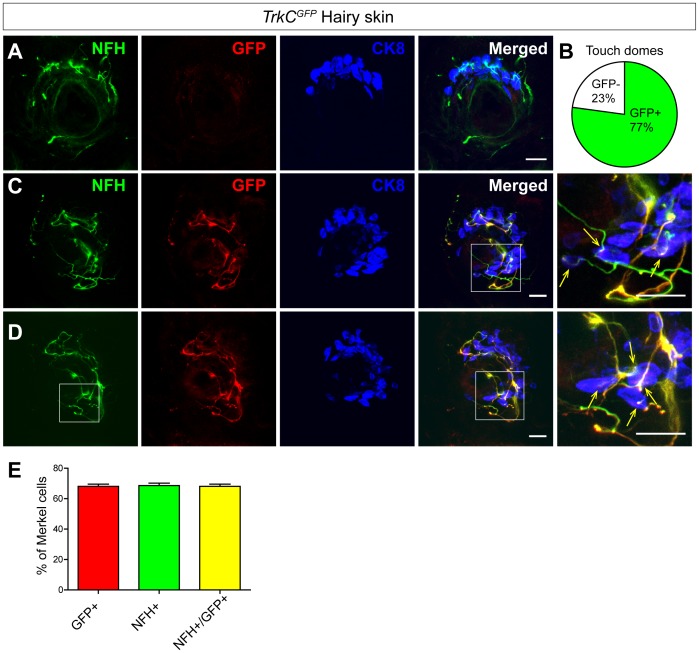
Merkel cell innervating NFH^+^ fibers are TrkC^+^. A, Triple staining of the back hairy skin of P0 *TrkC^GFP^* mice with antibodies against CK8, GFP and NFH showing one example of touch dome without GFP innervation. B, Pie chart showing that 77% of the touch domes are innervated by GFP^+^ fibers in P0 *TrkC^GFP^* mice. C-D, Two representative examples of touch domes with GFP^+^ fiber innervation. Almost all NFH^+^ fibers that innervate Merkel cells are GFP^+^, which are indicated by yellow arrows. E, Quantification of touch dome Merkel innervation by NFH^+^ and GFP^+^ fibers. Scale bar, A, C, D, 20 μm. n = 3 animals. See also Table 2.

To differentiate these two possibilities, we first asked if Merkel cell innervating NFH^+^ fibers are TrkC^+^. Given the close relationship of touch dome Merkel cell-neurite complexes and NT3/TrkC signaling [13], [15], [16], [26], it is widely believed that Merkel cell innervating neurites express TrkC. To confirm this idea, we took advantage of a BAC-EGFP transgenic line, *TrkC^GFP^* [22], [23], to directly visualize the TrkC^+^ nerve fibers. It was previously reported that this line labels ∼50% of TrkC^+^ DRG neurons neonatally [23]. Immunostaining of P0 *TrkC^GFP^* hairy skin with CK8, GFP, and NFH antibodies showed that ∼77% of the touch domes in back hairy skin are innervated by GFP^+^ fibers (Figure 2A-B and Table 2). In addition, within the GFP^+^ touch domes, 68.13±1.46% of the Merkel cells receive TrkC^+^ (stained with anti-GFP antibody) and NFH^+^ fiber innervation. Different from *Ret^CFP/+^* mice, in which some NFH^+^ and CFP^+^ signals are non-overlapping, NFH^+^ and GFP^+^ fibers in *TrkC^GFP^* mice almost completely overlap (Figure 2C-E and Table 2). This result provides direct evidence that most if not all NFH^+^ SAI mechanoreceptors, which innervate touch dome Merkel cells, are TrkC^+^. This result also suggests that the reason why CFP^+^ and NFH^+^ fibers in *Ret^CFP/+^* mice don’t completely overlap may not be due to the differential subcellular distribution of CFP and NFH.

**Table 2:** Touch dome Merkel cell innervation in P0 *TrkC^GFP^* hairy skin.

Genotype	# of TD	NFH^+^ TD (%)	GFP^+^ TD (%)	MC per TD	NFH^+^ MC (%)	GFP^+^ MC (%)	NFH^+^/GFP^+^ MC (%)	# of animal
*TrkC^GFP^*	70	70 (100%)	54 (77%)	20.16±1.284	14.00±1.087 (68.65±1.557%)	$13.88±1.°58 (68.13±1.458%)	13.88±1.058 (68.13±1.458%)	N = 3

$, only the touch domes with GFP innervation are used for quantification.

### Touch dome innervating Ret^+^ fibers depend on *TrkA* for their survival and normal innervation

To further determine that Ret^+^ and NFH^+^/TrkC^+^ fibers are two different types of afferents and to address whether Merkel cell innervating Ret^+^ fibers belong to the early or late Ret^+^ DRG neurons, we examined touch dome innervation in *TrkA* null mice. If these Ret^+^ fibers belong to the early Ret^+^ DRG neurons, their touch dome innervation should not be altered in *TrkA* knockout mice because early Ret^+^ neurons do not express *TrkA* and are independent of TrkA signaling [18]. On the other hand, if these Ret^+^ fibers belong to the late Ret^+^ DRG neurons, their innervation of touch dome Merkel cells would be greatly disrupted in *TrkA* null mice, as few late Ret^+^ DRG neurons survive in *TrkA* null mice [18], [20], [27].

**Figure 3:**
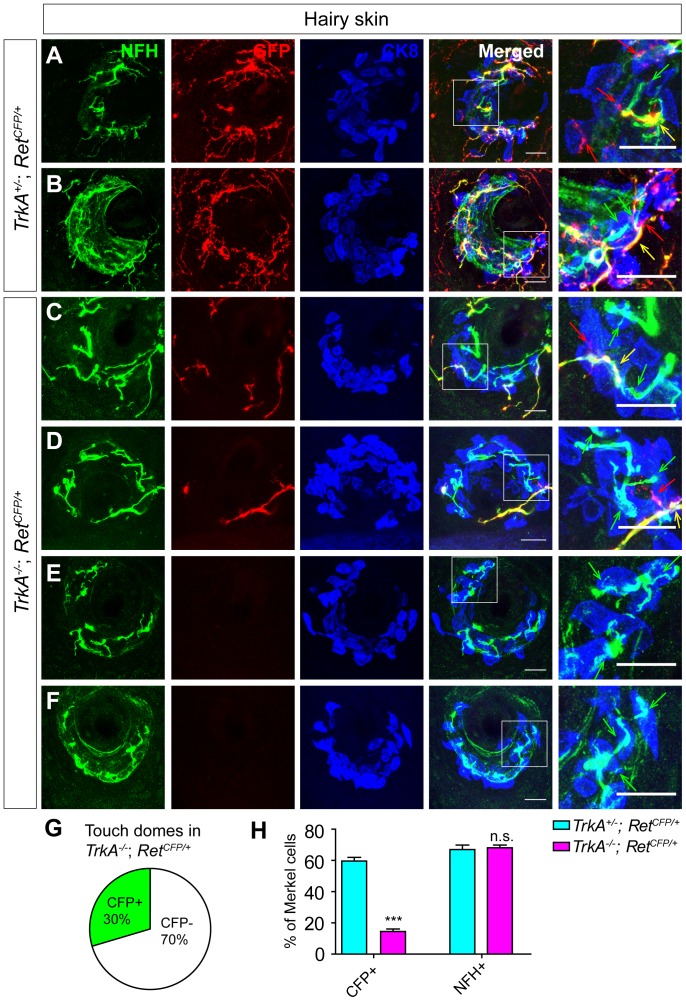
Most Merkel cell innervating Ret^+^ fibers are lost in *TrkA^−/−^* mice. A-B, Two examples of touch domes in P0 control *Ret^CFP/+^; TrkA^+/^* ^−^mice. The back hairy skin sections were triple stained with antibodies against CK8, GFP and NFH. Ret^+^ fibers are indicated by red arrows, NFH^+^ fibers are indicated by green arrows, and the overlap spots of Ret^+^ and NFH^+^ fibers are indicated by yellow arrows. C-D, Two representative examples of touch domes, which have greatly reduced CFP^+^ fiber innervation in P0 *Ret^CFP/+^;TrkA* ^−*/*−^ mutant mice. E-F, Two representative examples of touch domes, which completely lost CFP^+^ fiber innervation in P0 *Ret^CFP/+^; TrkA* ^−*/*−^ mutant mice. G, Pie chart showing that within the *Ret^CFP/+^; TrkA* ^−*/*−^ background, 70% of the touch domes have no CFP^+^ fiber innervation, while 30% of touch domes receive greatly decreased innervation of CFP^+^ fibers. H, Quantification of touch dome Merkel cell innervation by NFH^+^ (P = 0.8782) and CFP^+^ (P<0.001) fibers in *TrkA* mutant and control littermates. For *TrkA* mutant, only the 30% of touch domes with residual CFP^+^ fiber are counted and compared to the control. Scale bar, 20 μm. n = 4 for *Ret^CFP/+^; TrkA^+/^* ^−^ mice, and n = 5 for *Ret^CFP/+^; TrkA* ^−*/*−^ mice. ***P<0.001, n.s., no statistical significance. See also Table 3.

To test this idea, we generated *Ret^CFP/+^; TrkA^+^* ^/−^ control and *Ret^CFP/+^; TrkA* ^−/−^ mutant mice, in which Ret^+^ fibers are labeled with CFP in a *TrkA* null background. We found that Merkel cell innervating Ret^+^ fibers were significantly decreased in *TrkA* null mice, while the number of Merkel cells per touch dome section is comparable between mutant and control littermates (Figure 3, Table 3). 70% of the touch domes receive no CFP^+^ fiber innervation at all in *TrkA* mutant mice (Figure 3E-G). The remaining 30% of touch domes display dramatically reduced innervation of CFP^+^ fibers (Fig. 3C-D); with only 14.51±1.56% of total Merkel cells innervated by CFP^+^ fibers (59.67±2.31% in the control littermates) (Figure 3H). In contrast, the NFH^+^ fibers innervation is comparable between *TrkA* mutants (68.12±1.71%) and the littermate control (66.99±2.85%) (Figure 3H, Table 3), suggesting that NFH^+^ fibers are independent of TrkA signaling. These results indicate that the majority of Merkel cell innervating Ret^+^ fibers are dependent on TrkA signaling for their Merkel innervation, and thus belongs to the late Ret^+^ population. In addition, since almost all late Ret^+^ DRG neurons die in *TrkA* null mice [18], [20], [27], our results suggest that Ret^+^ and NFH^+^ fibers are two distinct populations of nerve fibers.

**Table 3:** Touch dome Merkel cell innervation in P0 *TrkA^+/^* ^−^ *; Ret^CFP/+^* control and *TrkA* ^−*/*−^ *; Ret^CFP/+^*mutant hairy skin.

Genotype	# of TD	NFH^+^ TD (%)	GFP^+^ TD (%)	MC per TD	NFH^+^ MC (%)	GFP^+^ MC (%)	NFH^+^/GFP^+^ MC (%)	# of animal
*TrkA^+/^* ^−^ *; Ret^CFP/+^*	50	50 (100%)	50 (100%)	21.44±1.424	15.40±1.292 (66.99±2.846%)	13.32±1.135 (59.67±2.314%)	11.64±1.078 (50.42±2.788%)	N = 4
*TrkA* ^−*/*−^ *; Ret^CFP/+^*	71	71 (100%)	21 (30%)	23.89 ±0.6480	16.35±0.6005 (68.12±1.712%)	$3.524±0.3818 (14.51±1.556%)	1.014±0.2156 (4.187±0.8916%)	N = 5
				n.s. P = 0.0877	n.s. P = 0.8782	*** P<0.001	*** P<0.001	

$ , only the touch domes with CFP innervation are used for quantification; ***, significant difference

### Touch dome innervation of Ret^+^ fibers is largely normal in *Ret* null mice

**Figure 4:**
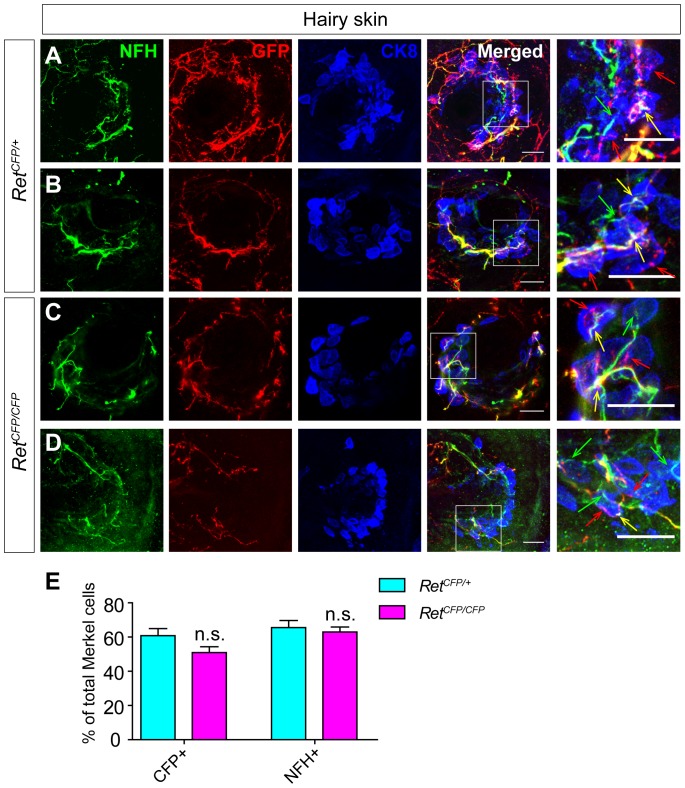
Merkel cell innervating Ret^+^ fibers show subtle phenotype in *Ret^CFP/CFP^* mice. A-B, Two examples of touch domes in control P0 *Ret^CFP/+^* mice. The back hairy skin sections were triple stained with antibodies against CK8, GFP and NFH. Ret^+^ fibers are indicated by red arrows, NFH^+^ fibers are indicated by green arrows, and the overlap spots of Ret^+^ and NFH^+^ fibers are indicated by yellow arrows. C-D, Two examples of touch domes in P0 *Ret^CFP/CFP^* null mice. E, Quantification of touch dome Merkel innervation by NFH^+^ (P = 0.5723) and CFP^+^ (P = 0.1635) fibers in *Ret* null and control littermates. Scale bar, 20 μm. n = 3 for both genotypes. See also Table 1.

To determine the role of Ret signaling in controlling development of Merkel cell-neurite complex in touch domes, we generated *Ret^CFP/CFP^* null mice, in which both copies of the *Ret* gene are knocked out while CFP is expressed to mark all Ret^+^ cells. We observed a trend of decrease of touch dome innervating CFP^+^ fibers in *Ret^CFP/CFP^* null mice, while the Merkel cell number per touch dome was not affected. However, this slight difference is not a statistically significant between *Ret* mutant (51.00±3.38%) and control littermates (60.81±4.19%) (Figure 4A-E, Table 1, P = 0.1635), indicating that Ret signaling plays a minor role in this developmental process. In addition, the NFH^+^ fiber innervation was indistinguishable between *Ret* mutant (62.95±2.93%) and control littermates (65.59±4.11%) (Figure 4E, Table 1). Taken together, our results suggest that touch dome Merkel cell innervating Ret^+^ fibers belong to the late Ret^+^ neurons, which rely on TrkA but not Ret signaling for their neonatal Merkel cell innervation.

#### Merkel cells in the neonatal glabrous skin are mainly innervated by NFH+/TrkC+ fibers

**Figure 5:**
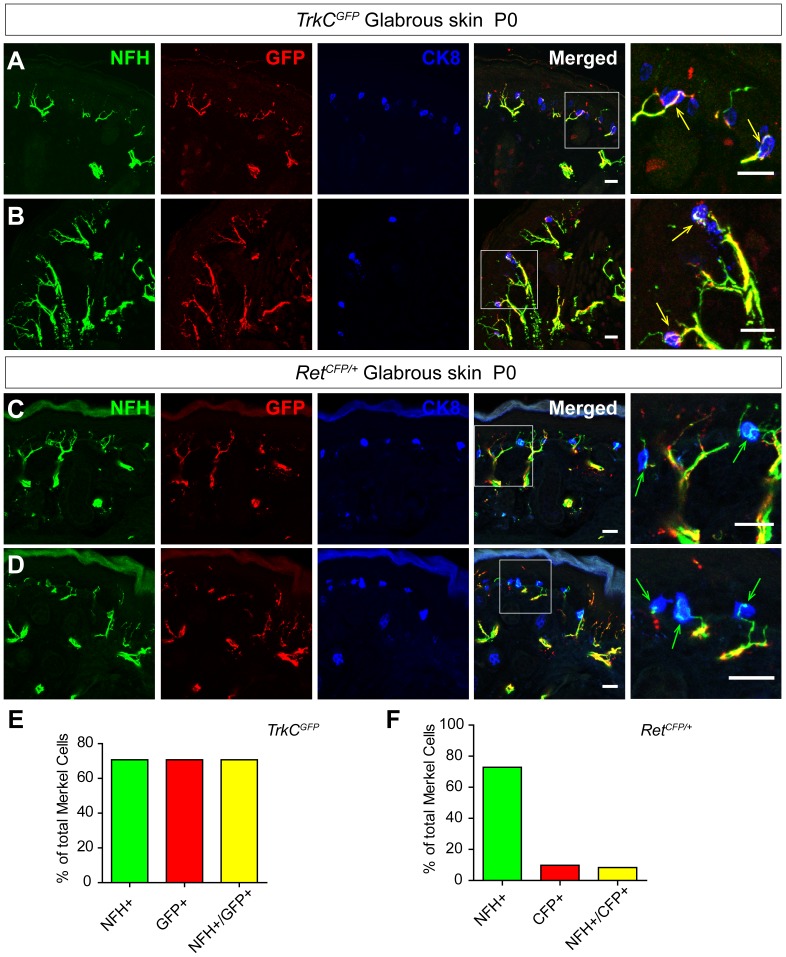
Majority of the Merkel cells in the neonatal glabrous skin are innervated by TrkC^+^ but not Ret^+^ fibers. A-B, Two examples of Merkel cell innervation in the glabrous skin of P0 *TrkC^GFP^* mice. These glabrous skin sections were triple stained with antibodies against CK8, GFP and NFH. Almost all GFP^+^ Merkel cell innervating fibers overlap with NFH^+^ fibers, which are indicated by yellow arrows. C-D, Two examples of Merkel cell innervations in the glabrous skin of P0 *Ret^CFP/+^* mice. Most of the Merkel cells are innervated by NFH^+^ rather than CFP^+^ fibers. E-F, Quantification of Merkel innervation in the glabrous skin by NFH^+^/TrkC^+^ or Ret^+^ fibers in *TrkC^GFP^* mice (G) and *Ret^CFP/+^* mice (H). Scale bar, 20 μm, n = 3 for both genotypes. See also Table 4.

Do Merkel cells in the glabrous skin also receive dual innervation? To answer this question, we examined the Merkel cell-neurite complexes of hind paw foot pads in both *Ret^CFP/+^* and *TrkC^GFP^* mice. We found that Merkel cells, which reside along the epidermis/dermis boundary in the dermal papilla, are innervated by NFH^+^/GFP^+^ fibers in *TrkC^GFP^* mice (Figure 5A-B, green arrows). ∼70% of Merkel cells are innervated and all of them receive NFH^+^/GFP^+^ fiber innervation in *TrkC^GFP^* mice (Figure 5E, Table 4). In contrast, only a small percentage of Merkel cells (9.75%) are innervated by CFP^+^ fibers in *Ret^CFP/+^* mice (Figure 5C-D, F, Table 4), suggesting that most neonatal Merkel cells in the glabrous skin receive TrkC^+^ but not dual innervation.

**Table 4:** Glabrous skin Merkel cell innervation at P0.

Genotype	# of MC	NFH^+^ MC (%)	GFP^+^ MC (%)	NFH^+^/GFP^+^ MC (%)	# of animal
*TrkC^GFP^*	165	117 (70.91%)	117 (70.91%)	117 (70.91%)	N = 3
*Ret^CFP/+^*	166	120 (72.85%)	15 (9.75%)	13 (8.34%)	N = 3

#### Merkel cells in the adult touch domes receive both Ret+ and NFH+ fiber innervation

**Figure 6:**
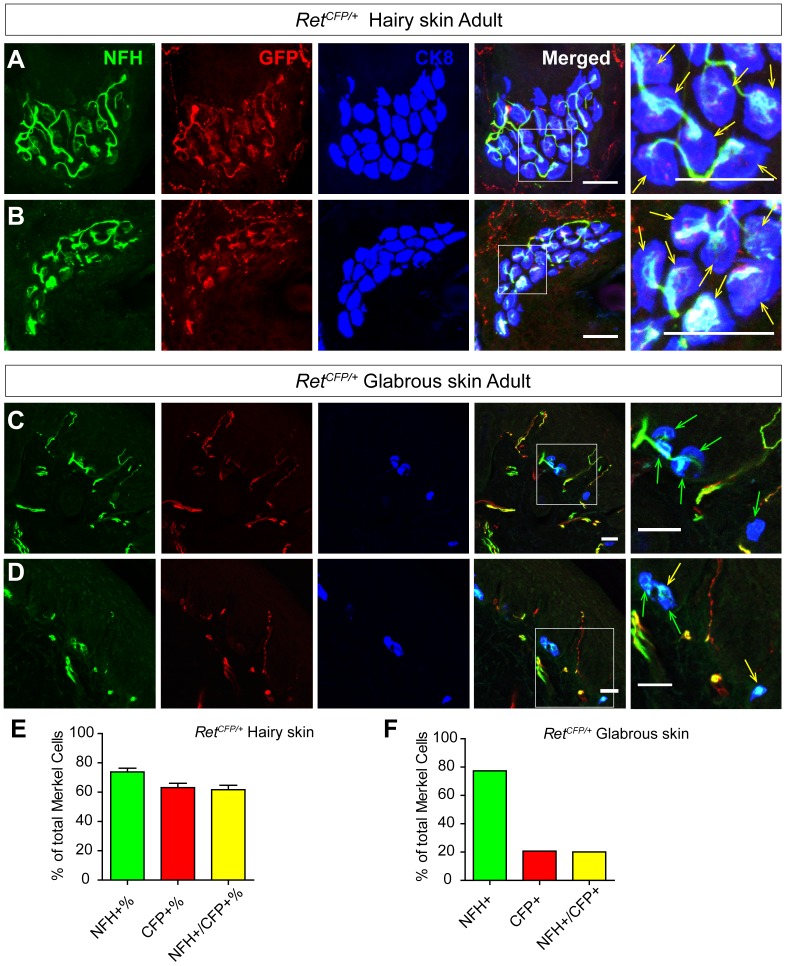
Adult Merkel cells receive NFH^+^ and Ret^+^ fiber innervation. A-B, Triple staining of the back hairy skin of adult *Ret^CFP/+^* mice with antibodies against CK8, GFP and NFH. Most of the Merkel cells in the touch dome are innervated by both NFH^+^ and CFP^+^ fibers, which are indicated by yellow arrows. NFH and GFP signals largely overlap upon the innervation area of Merkel cells. C-D, Glabrous skin staining of adult *Ret^CFP/+^* mice showing that most Merkel cells are innervated by NFH^+^ fibers. Merkel cells innervated by NFH^+^ fibers are indicated by green arrows while those innervated by both NFH^+^ and CFP^+^ fibers are indicated by yellow arrows. E-F, Quantification of Merkel cells innervated by NFH^+^ and CFP^+^ fibers in adult trunk hairy skin (E) and glabrous skin (F). Scale bar, 20 μm, n = 3. See also Table 5 and 6.

To determine if Ret^+^ fibers persistently innervate the touch dome Merkel cells, we examined back hairy skin sections of adult *Ret^CFP/+^* mice using triple staining of CK8, GFP, and NFH. We found that 61.74±3.01% of adult touch dome Merkel cells receives both NFH^+^ and CFP^+^ fiber innervation (Figure 6E, Table 5). We also noticed that the adult innervation pattern is somehow different from neonatal staining. Mature Merkel cell-neurite complexes showed synaptic bouton-like structures in the touch dome, and CFP^+^ and NFH^+^ fibers overlap much better in the adult hairy skin (Figure 6A-B, Table 6). In the adult glabrous skin of the hind paw, we found that most of the Merkel cells are still innervated by NFH^+^ fibers (77.41%, Figure 6C, D and F, Table 6) while slight more Merkel cells (20.67%) are innervated by CFP^+^ fibers comparing with P0.

**Table 5:** Touch dome Merkel cell innervation in adult *Ret^CFP/+^* mice.

Genotype	# of TD	NFH+ TD (%)	GFP+ TD (%)	MC per TD	NFH+ MC (%)	GFP+ MC (%)	NFH+/GFP+ MC (%)	# of animal
*Ret^CFP/+^*	24	24 (100%)	24 (100%)	18.88±1.784	14.08±1.432 (73.75±26.35%)	12.00±1.242 (63.11±2.953%)	11.75±1.227 (61.74±3.006%)	N = 3

**Table 6:** Glabrous skin Merkel cell innervation in adult *Ret^CFP/+^* mice.

Genotype	# of MC	NFH^+^ MC (%)	GFP^+^ MC (%)	NFH^+^/GFP^+^ MC (%)	# of animal
*Ret^CFP/+^*	230	180 (77.41%)	49 (20.67%)	48 (20.14%)	N = 3

**Figure 7:**
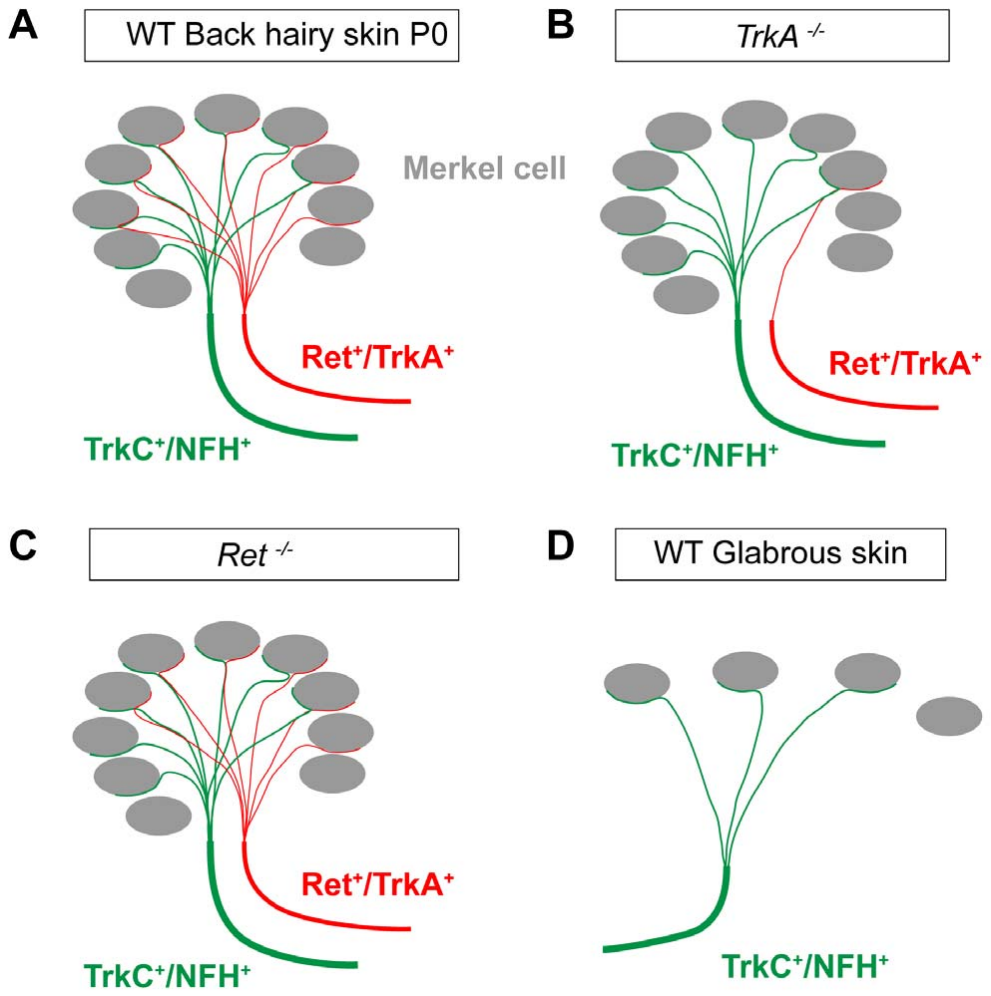
Schematic views of Merkel cell innervation in wild type, *TrkA^−/−^* and *Ret^−/−^* neonatal touch domes and the wild type glabrous skin. A, In the trunk hairy skin of wild type mouse, touch dome Merkel cells are innervated by two types of fibers: NFH^+^/TrkC^+^ and Ret^+^/TrkA^+^ fibers. Most Merkel cells receive dual innervation from both types. B, In *TrkA^−/−^* mice, most Ret^+^/TrkA^+^ fibers are lost, while NFH^+^/TrkC^+^ fibers are not affected in the touch dome innervation. C, In the back hairy skin of *Ret^−/−^* mice, Ret^+^ and NFH^+^ fibers are not significantly changed. D, In the glabrous skin of wild type mouse, Merkel cells are mainly innervated by NFH^+^/TrkC^+^ fibers.

Taken together, our results provide evidence that most Merkel cells in mouse neonatal touch domes receive dual innervation of Ret^+^/TrkA^+^ and NFH^+^/TrkC^+^ fibers (Figure 7A). These Ret^+^ innervating fibers belong to the late but not early Ret^+^ neurons since they depend on TrkA signaling for their prenatal development (Figure 7B-C). In adult mice, touch dome Merkel cells are still innervated by both Ret^+^ and NFH^+^ fibers. In contrast, Merkel cells in the glabrous skin mainly receive innervation of NFH^+^/TrkC^+^ fibers (Figure 7D).

## Discussion

### Dual innervation of touch dome Merkel cells in the hairy skin

In rodents, Merkel cells are located in whisker follicles, touch domes of back hairy skin, and glabrous skin of the foot pads. The whiskers are very sensitive and specialized tactile organs, and contain large numbers of Merkel cells and associated nerve endings. It has been well demonstrated that there are at least two sets of Merkel cell innervation fibers in the prenatal whisker pad [10], [11], [28]: one set initially develops in response to either NGF or NT3 signaling through TrkA, whereas the other set depends upon TrkC. Both sets become TrkC dependent postnatally. However, it is unclear whether this is also true for the trunk hairy skin and glabrous skin, since Merkel cells in the whisker follicles are innervated by trigeminal ganglion neurons, while Merkel cells in the trunk hairy skin and glabrous skin are innervated DRG neurons.

Using genetic tracing and neurotrophic factor dependence analysis, here we provide evidence that neonatal touch dome Merkel cells are also innervated by two types of afferents: one expresses Ret and the other expresses NFH and TrkC (Figure 1, 2, and 7A). Interestingly, Ret^+^ but not NFH^+^/TrkC^+^ fibers highly depend on TrkA signaling for innervation (Figure 7B): 70% of the touch domes completely lost their Ret^+^ fiber innervation in *TrkA* null mice, and the other 30% touch domes lost about 86% of Ret^+^ fiber innervation (Figure 3). The loss of Ret^+^ fibers in *TrkA* null mice are most likely due to cell death since almost all late Ret^+^ DRG neurons die in *TrkA* null mice [18], [20], [27]. Though the expression of *Ret* in the late Ret^+^ DRG neurons is also dependent on NGF/TrkA signaling, the cell death phenotype is much more dominant and the gene regulation effect of NGF/TrkA signaling can only be revealed when neuronal apoptosis is blocked [18], [29]. Thus, our results strongly suggest that neonatal Merkel cells receive innervation of two distinct populations of nerve fibers.

Given that NFH is usually expressed in highly myelinated fibers [30], these Ret^+^/TrkA^+^ touch dome innervating fibers are likely to be unmyelinated or thinly myelinated. Thus, our results suggest that neonatal touch dome Merkel cells may receive innervation other than the highly myelinated SAI mechanoreceptors. It is interesting that neonatal Merkel cells in both whisker pads and the trunk hairy skin receive dual innervation, suggesting that Merkel cell-neurite complexes around hair follicles may function as multimodal sensory receptors [31].

Touch dome Merkel cells in adult mice also receive both Ret^+^ and NFH^+^ fibers innervation. However, CFP^+^ and NFH^+^ signals largely overlap upon the innervation of individual Merkel cells (Figure 6A-B), which is quite different from the neonatal pattern. At present, we can’t conclude if adult Merkel cell innervating Ret^+^ and NFH^+^ fibers belong to the same or different populations of DRG neurons. It is possible that the adult touch dome Merkel cells receive the same dual innervation as neonatal mice. Ret^+^ and NFH^+^ fibers may overlap much better in adult touch domes simply due to postnatal maturation. On the other hand, it is also possible that *Ret* is expressed in adult but not neonatal NFH^+^ SAI mechanoreceptors. Unfortunately, the *TrkA* null mice die at P0, so we can’t differentiate these two possibilities in this paper. Future studies are necessary to determine the nature of these Merkel cell innervating Ret^+^ fibers in adult mice.

On the other hand, most Merkel cells in the mouse foot pad glabrous skin only receive one types of innervation (TrkC^+^/NFH^+^) (Figure 5, 6C-E and 7D). Although previous publication suggests that Ret^+^ fibers do not innervate glabrous skin Merkel cells [19], we did find a small percentage of foot pad Merkel cells that are innervated by Ret^+^ fibers. This discrepancy could be explained by the different methods. The previous paper used Ret antibody staining [19] while we used *Ret^CFP^* knockin mice to reveal Ret^+^ fibers in the skin. Interestingly, Merkel cells in the hairy and glabrous skin also respond differently to neonatal denervation [32], suggesting that Merkel cells in the hairy and glabrous skin are somehow different. At present, it is unclear if this innervating nerve fiber difference induces Merkel cells in the hairy and glabrous skin to be different or the differences between Merkel cells in the hairy and glabrous skin attract different types of innervating fibers. In addition, the functional consequence of this Merkel cell-neurite complex difference between the hairy and glabrous skin remains elusive.

### Late but not Early Ret^+^ DRG neurons innervate Merkel cells

Using genetic labeling of Ret^+^ nerve fibers and *TrkA* null mice, we showed that the majority of Ret^+^ fibers that innervate touch dome Merkel cells belong to the late but not early Ret^+^ neurons. This conclusion is drawn based on: 1) Ret^+^ and NFH^+^ fibers do not completely overlap (Figure 1); and 2) Ret^+^ fibers highly depend on TrkA but not Ret signaling for their survival and Merkel cell innervation (Figure 4). This is consistent with the previous observation that in the late Ret^+^ neurons, NGF/TrkA signaling governs most prenatal developmental processes, such as the embryonic survival and expression of genes initiated before P0, while Ret signaling gradually takes over after birth to control the epidermal innervation and expression of genes initiated postnatally [17], [18], [20].

Although previous work [19] showed that Ret^+^/NFH^+^ fibers innervate touch dome Merkel cells using double immunostaining, it is hard to tell whether Ret^+^ and NFH^+^ fibers are the same afferents or they are different afferents but bundling together, based on the resolution of published images. In addition, the subcellular location of Ret (a membrane protein) and CFP (a cytosol protein) may be different, which could contribute to the slightly different staining pattern of Ret/NFH and CFP/NFH. Importantly, our analysis of *TrkA* null mice provides a piece of strong evidence that the neonatal Merkel cell innervating Ret^+^ and NFH^+^ fibers are two different types of fibers because most Ret^+^ fibers are lost in *TrkA* null mice but NFH^+^ fibers are unaltered (Fig. 3). Another noticeable difference between this and the previous study [19] is that we found no significant decrease of NFH^+^ Merkel cell innervating fibers in *Ret* null mice (Figure 4 and 7C), which may be explained by the different quantification methods. The previous publication was based on quantifying the averaged area of NFH^+^ signal within the touch domes [19] while we counted the number and percentage of Merkel cells that receive CFP^+^ or NFH^+^ fiber innervation. Some change of NHF^+^ fibers may lead to a reduction in total innervation area, without causing a change in the percentage of Merkel cells innervated by NFH^+^ fibers.

In addition to NTRK signaling, recent studies revealed the involvement of different transcription factors, such as Shox2 and Runx3, in determining the development of Merkel cell innervating afferents [33], [34]. Moreover, genetic tracing of VGluT3^+^ DRG neurons indicated that some sensory neurons, which transiently express VGluT3, are myelinated mechanoreceptors innervating Merkel cells [35]. Given our finding that neonatal touch dome Merkel cells receive dual innervations, future studies are necessary to determine if the identified regulatory mechanisms control development of only one or both types of fibers.

### Conclusion

1. Neonatal touch dome Merkel cells receive dual innervation: one population of fibers is Ret^+^/TrkA^+^ and the other population is NFH^+^/TrkC^+^.

2. Based on their high dependence on TrkA signaling, the majority of Ret^+^ fibers innervating neonatal touch dome Merkel cells belong to the late but not early Ret^+^ neurons.

3. Adult touch dome Merkel cells are innervated by Ret^+^ and NFH^+^ fibers.
